# Case Report: Unveiling an Incidentally Diagnosed Extrapulmonary Small Cell Carcinoma of the Rectum

**DOI:** 10.7759/cureus.46920

**Published:** 2023-10-12

**Authors:** Sagar Nagpal, Amro Daoud, Katrina A Taylor, Mohammad A Parvez, Jason Mckinney

**Affiliations:** 1 Department of Internal Medicine, East Tennessee State University Quillen College of Medicine, Johnson City, USA; 2 Division of Gastroenterology, East Tennessee State University Quillen College of Medicine, Johnson City, USA; 3 Department of Pathology, East Tennessee State University Quillen College of Medicine, Johnson City, USA

**Keywords:** colorectal cancer, histopathological diagnosis, extrapulmonary small cell cancer, rectal neuroendocrine tumor, small cell rectal cancer

## Abstract

Extrapulmonary small cell carcinoma (EPSCC) is a rare malignancy with distinct clinical and pathological characteristics. We present the case of a 72-year-old male diagnosed with EPSCC of the rectum during a routine screening colonoscopy. The patient was asymptomatic, and pathological examination revealed a rectal mass displaying features of small cell carcinoma (SCC) associated with tubular adenoma. The treatment comprised radiation therapy and cisplatin/etoposide chemotherapy. This case underscores the importance of considering EPSCC as a potential diagnosis in patients with rectal masses, necessitating further studies to optimize treatment strategies.

## Introduction

Small cell carcinomas (SCCs) are a distinctive category within the realm of poorly differentiated tumors, encompassing entities, such as small cell lung cancer and extrapulmonary small cell cancer (EPSCC). Although EPSCC represents a relatively rare occurrence, it demonstrates a propensity for appearing in diverse anatomical locations across the body, with a predilection for the gastrointestinal tract [1]. While EPSCC is relatively uncommon, it tends to manifest in various parts of the body, with a particular preference for the gastrointestinal tract [1]. EPSCC accounts for roughly 0.1% to 0.4% of all cancer cases, equating to approximately 1000 new diagnoses each year [2,3]. In the gastrointestinal tract, the esophagus is the primary location for EPSCC (53% of cases), followed by the colon (13%) and the stomach (11%) [4]. EPSCC's notable characteristic lies in its aggressive behavior, often leading to intricate clinical courses. However, the understanding of EPSCC's prognosis and optimal treatment strategies remains limited due to the scarcity of comprehensive data stemming from its infrequent presentation. The aggressive nature of this unique subset underscores the necessity for a more profound understanding, particularly due to the absence of standardized therapeutic protocols. This differentiation from the extensively studied small cell lung cancer adds complexity to the management of EPSCC.

## Case presentation

**Figure 1:**
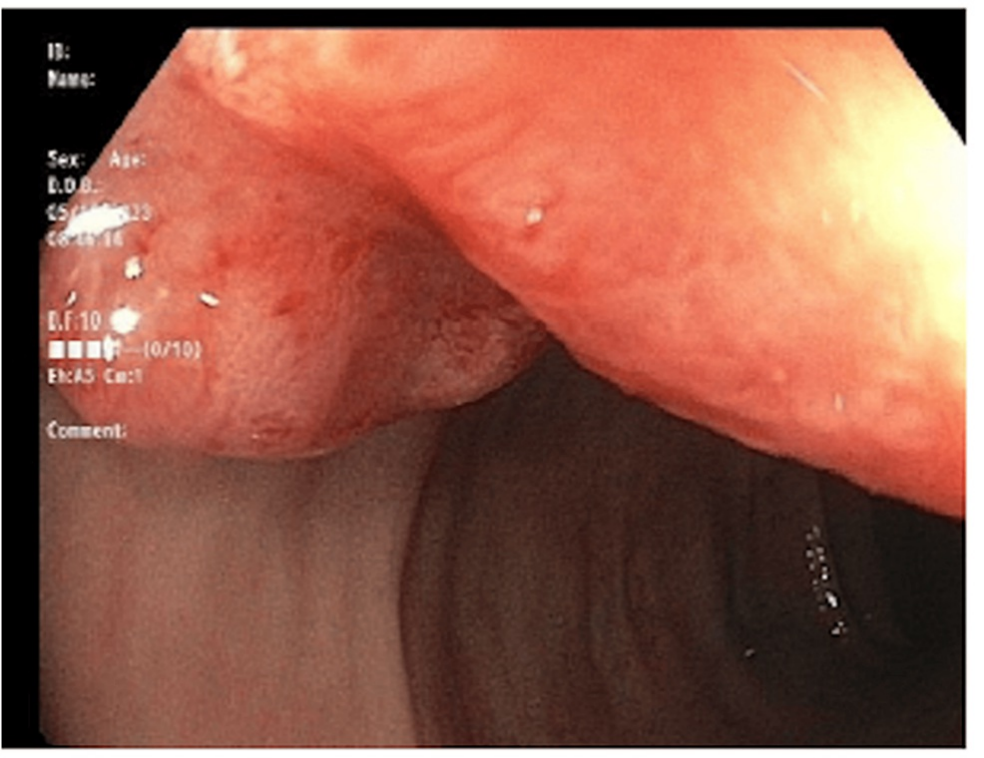
Colonoscopy picture showing a fungating rectal mass with central umbilication and ulceration.

**Figure 2:**
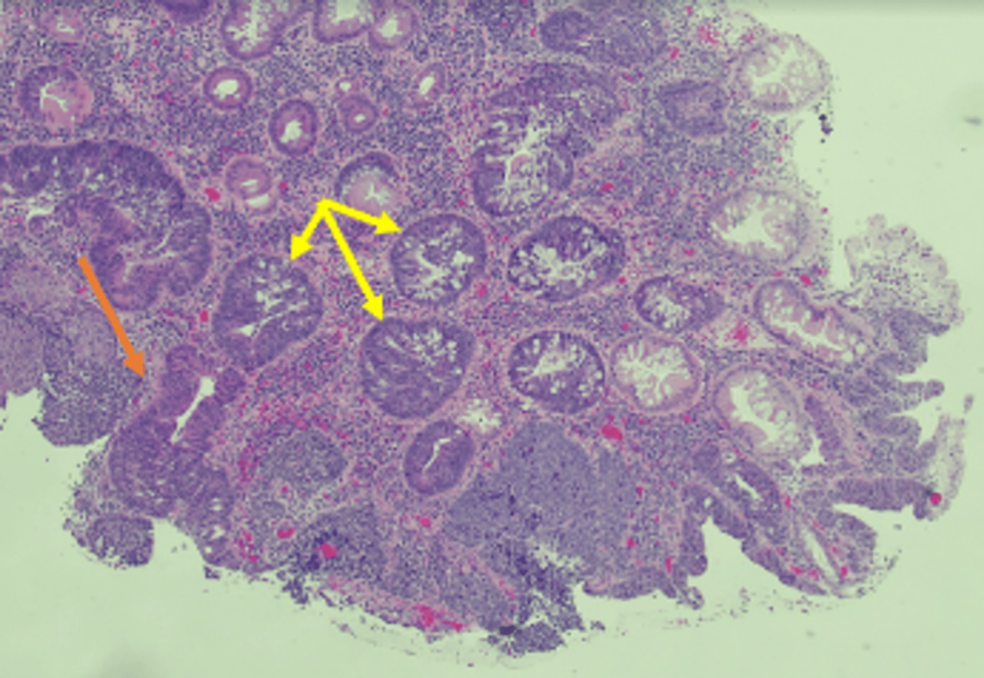
Rectal mass specimen. H&E (hematoxylin & eosin) of the biopsy shows sheets of small to medium round blue cells with a minimal cytoplasm interspersed between colonic glands. The glandular dysplasia ranges from low (tubular adenoma) to high (carcinoma in situ).

**Figure 3:**
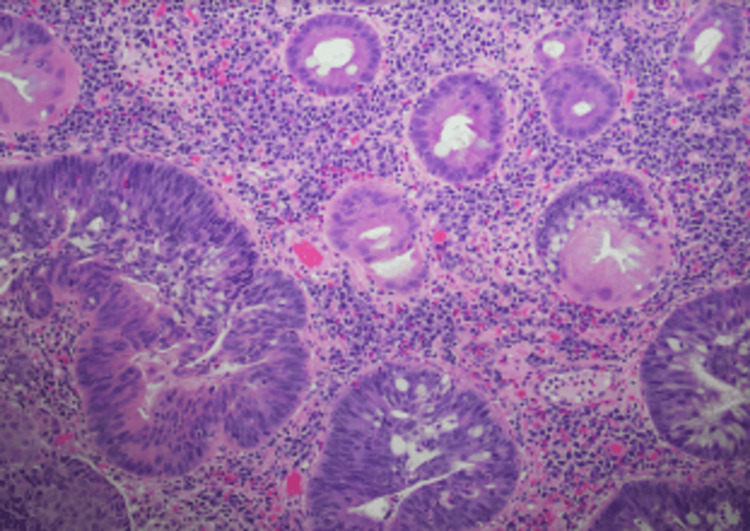
Rectal mass specimen. H&E (hematoxylin & eosin) of the biopsy showing nuclear features of small cell carcinoma including finely dispersed chromatin, no distinct nucleoli, high nuclear-to-cytoplasmic ratio, and high mitotic rate.

**Figure 4:**
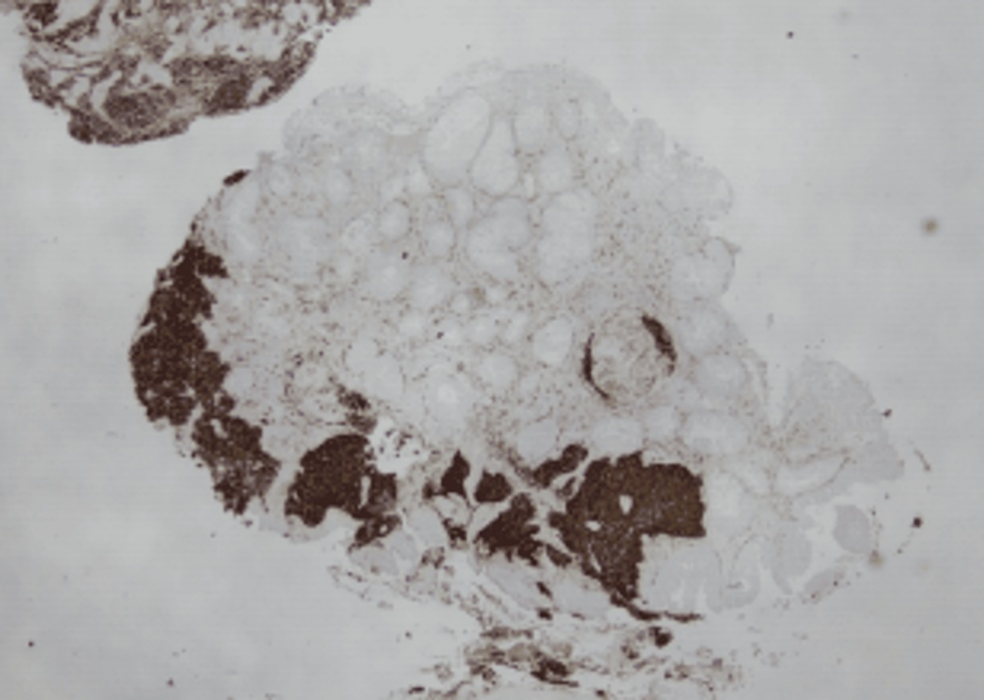
Immunoperoxidase stains show the tumor to be positive for CD56.

**Figure 5:**
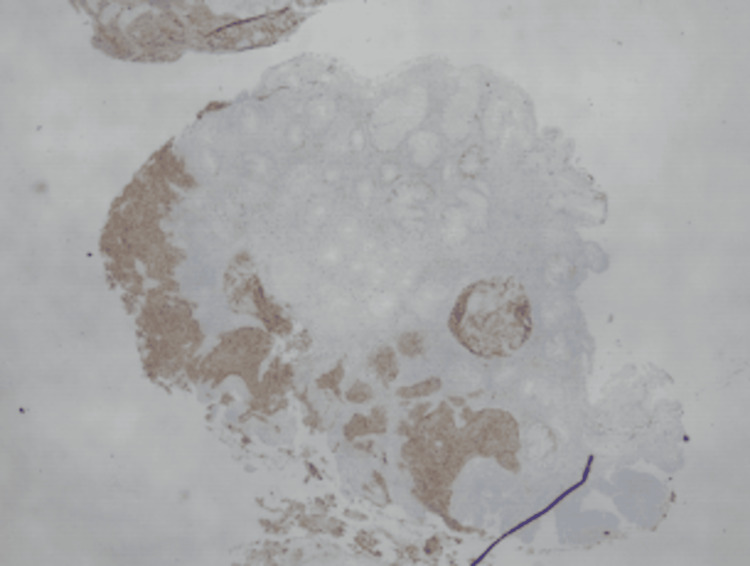
Immunoperoxidase stains show the tumor to be positive for synaptophysin.

A 72-year-old male with a significant medical history of type 2 diabetes mellitus presented for a screening colonoscopy. His social history revealed exposure to Agent Orange and employment at a nuclear facility, while no history of tobacco smoking or alcohol use was reported. During the colonoscopy, a rectal mass measuring approximately 3 cm in size, located 20 cm from the anal verge, was discovered (Figure 1). The mass exhibited central umbilication and ulceration features. The subsequent biopsy confirmed the presence of SCC associated with tubular adenoma exhibiting high-grade glandular dysplasia/carcinoma in situ. In addition, nuclear features characteristic of SCC, including finely dispersed chromatin, absence of distinct nucleoli, high nuclear-to-cytoplasmic ratio, and high mitotic rate, were observed (Figures 2, 3). Immunohistochemical analysis showed positive staining for CD56 and synaptophysin (Figures 4, 5), while chromogranin, SATB2, CDX2, and CK20 stains were negative.

**Figure 6:**
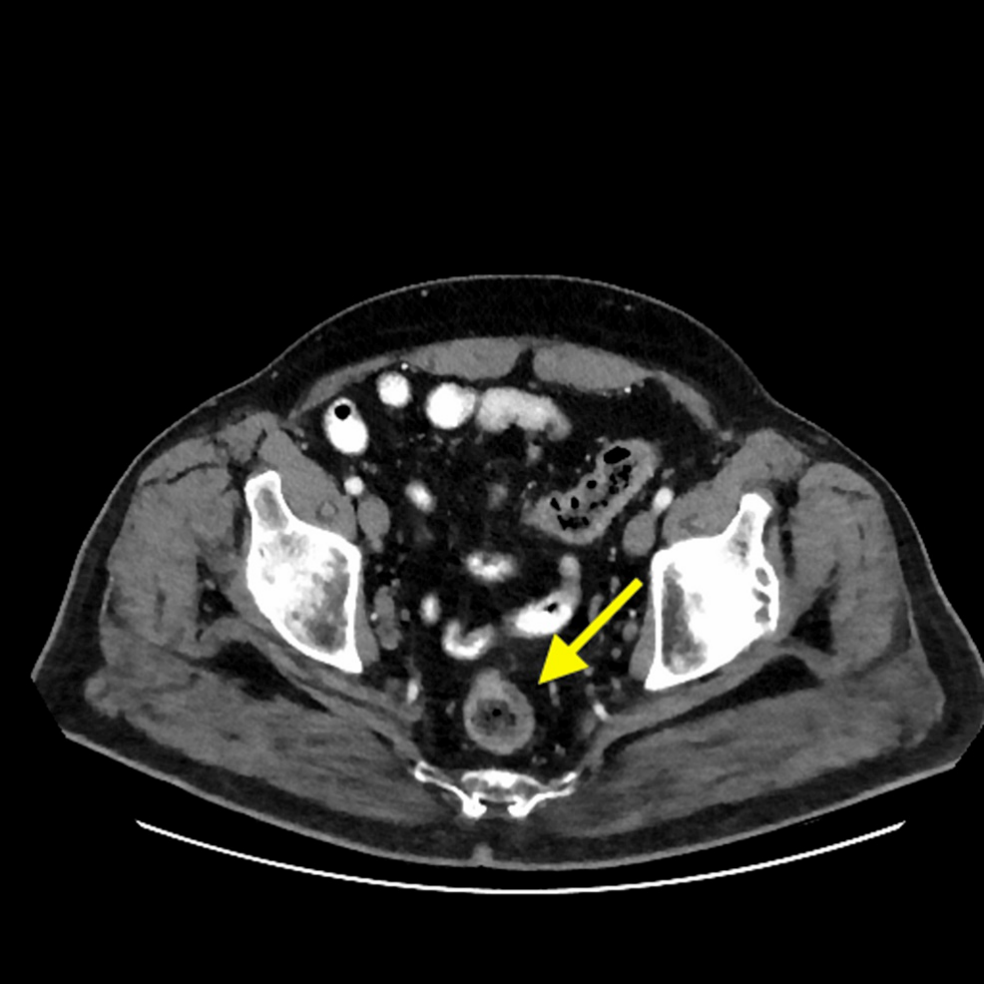
Cross-sectional image of the computed tomography (CT) of the abdomen showing rectal wall thickening

**Figure 7:**
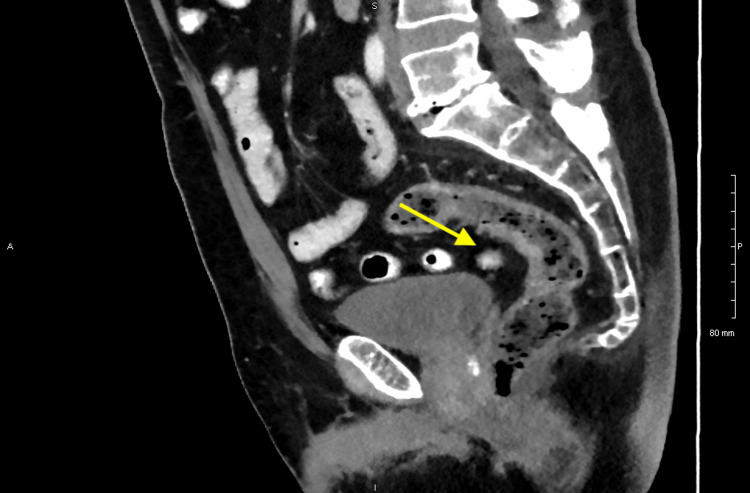
Computed tomography (CT) scan of the abdomen showing a necrotic perirectal lymph node.

**Figure 8:**
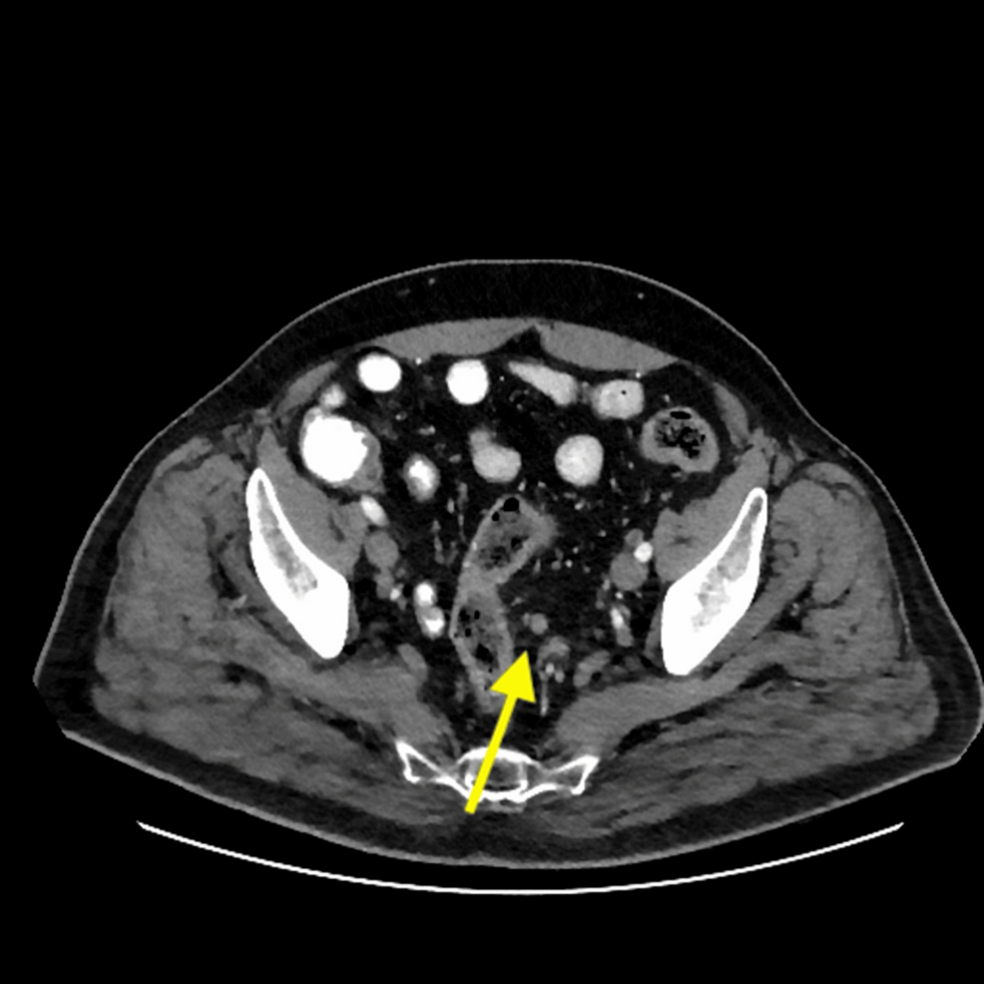
Computed tomography (CT) scan of the abdomen showing a prominent perirectal lymph node.

A computed tomography (CT) scan of the chest, abdomen, and pelvis revealed thickening along the rectum, potentially associated with the suspected mass (Figure 6). Furthermore, necrotic perirectal lymph nodes were detected, raising suspicion of metastasis (Figures 7, 8).

The patient received the treatment with cisplatin and etoposide. On a follow-up positron emission tomography (PET) scan after four months, no metabolically active lymphadenopathy or any active lesion in the rectum was seen. The patient tolerated the treatment very well and reported no symptoms at the follow-up visit after five months.

## Discussion

Treatment options for EPSCC are akin to those used for SCC of the lung, with concurrent platinum-based chemotherapy and radiation being the standard for limited-stage disease.

The patient's diagnosis of EPSCC of the rectum is a rare malignancy. EPSCC diagnosis relies on the presence of SCC attributes, outside the lung. Notably, this malignancy predominantly targets individuals in the middle to elderly age group, with over 70% of diagnoses occurring after the age of 50 [5]. EPSCC has similar characteristics to small cell lung cancer on histology, which includes inconspicuous nucleoli, round- to spindle-shaped small cells with dense nuclei, and sparse cytoplasm. Both epithelial and primitive neuroendocrine differentiations are seen in immunohistochemistry, often staining it positive for chromogranin A and synaptophysin [1]. Studies suggest that fluorodeoxyglucose (FDG) PET is useful in both staging and restaging of EPSCC. EPSCC is highly PET avid like SCC of the lung. PET CT can influence the management by identifying additional sites of disease [6].

Although our knowledge in this domain is constrained, some studies hint at similar response rates to chemotherapy and radiotherapy, when compared to surgical interventions [5]. However, the landscape is diversified, with varying reports on the benefits of surgery, ranging from moderate advantages to no significant benefits in comparison to combined chemotherapy and radiotherapy approaches. 

The prognosis of EPSCC is dependent on the tumor stage and site. Moreover, the prognosis of EPSCC hinges on the site of malignancy, with gastrointestinal EPSCC showing the worst outcomes and breast EPSCC the best [7]. In a study conducted by Brenner et al., it was noted that gastrointestinal (GI) small cell cancers, regardless of whether they were situated in the upper or lower GI tract, displayed similar clinicopathological features. Furthermore, their responses to chemotherapy and the rates of progression were observed to be consistent across these anatomical locations [8]. The spectrum of median survival in gastrointestinal EPSCC spans from mere weeks to six to 12 months, with certain investigations even noting survival around the 14-month mark [9]. Studies emphasize that a considerable 56% of patients with GI EPSCC exhibit extensive disease at the point of diagnosis [10].

## Conclusions

Navigating the management of EPSCC often draws on guidelines developed for small cell lung cancer, given the dearth of tailored directives. This absence of specific recommendations underscores the need for comprehensive research in EPSCC management and prognosis.

As this report delves into the multifaceted landscape of EPSCC, it aspires to deepen our grasp of its clinical characteristics, optimal management modalities, and the overall projection of outcomes.

Further research is imperative to better understand the management and prognosis of EPSCC of the rectum due to limited available data. In addition, investigating potential associations between radiation and Agent Orange exposure and EPSCC is warranted. Accurate histologic diagnosis plays a vital role and can significantly impact the management approach for this rare malignancy.
